# Two new species of the genus *Trilacuna* Tong & Li, 2007 (Araneae, Oonopidae) from the Nangunhe Nature Reserve, Yunnan Province, China

**DOI:** 10.3897/BDJ.13.e175820

**Published:** 2025-11-25

**Authors:** Chengzhi Dai, Tong Yang, Yanfeng Tong, Zizhong Yang

**Affiliations:** 1 College of Life Science, Shenyang Normal University, Shenyang 110034, China College of Life Science, Shenyang Normal University Shenyang 110034 China; 2 Yunnan Nangunhe National Nature Reserve Management and Protection Bureau, Cangyuan 677400, China Yunnan Nangunhe National Nature Reserve Management and Protection Bureau Cangyuan 677400 China; 3 National-Local Joint Engineering Research Center of Entomoceutics, Dali University, Dali 671000, China National-Local Joint Engineering Research Center of Entomoceutics, Dali University Dali 671000 China

**Keywords:** morphology, taxonomy, goblin spiders

## Abstract

**Background:**

The spider genus *Trilacuna* Tong & Li, 2007, currently comprises 32 species known from China, of which 15 species are from Yunnan and one species, i.e. *T. wumanshan* Tong, Yang & Zhang, 2023 is recorded from the Nangunhe Nature Reserve, Yunnan.

**New information:**

Two new *Trilacuna* species, *Trilacuna jimengi* Dai & Tong, sp. nov. (♂♀) and *Trilacuna nangunhe* Dai & Tong, sp. nov. (♂♀), are described, based on specimens collected from the Nangunhe Nature Reserve, Yunnan Province. Morphological descriptions and photomicroscopy images of the new species are given.

## Introduction

The spider family Oonopidae, commonly known as goblin spiders, represents a highly diverse lineage comprising 1978 extant species in 115 genera (WSC 2025). The genus *Trilacuna* was established, based on two new species collected from Yunnan and Chongqing, China (Tong and Li 2007). The genus *Trilacuna* currently comprises 53 valid species, distributed from Iran to the Korean Peninsula and south to Sumatra, Indonesia (Dai et al. 2025).

The Nangunhe Nature Reserve is a 708 km^2^ protected area, located in western Cangyuan County, Yunnan Province, China (23°15.00′N, 99°0.00′E). Nangunhe is located in the northernmost fringe of the Southeast Asian tropical zone and situated in the middle of the Myanmar China border. It is one of China’s priority conservation areas established in 1980 (Bohnett et al. 2015). One species of the genus *Trilacuna* has been recorded in Nangunhe, i.e. *T. wumanshan* Tong, Yang & Zhang, 2023 (Ma et al. 2023) before this study.

In this paper, two new species of *Trilacuna* are described from the Nangunhe Nature Reserve. Detailed morphological descriptions, diagnostic photographs and a distribution map are given.

## Materials and methods

The specimens were examined under a Leica M205 C stereomicroscope. Fine details were studied under an Olympus BX51 compound microscope. Endogynes were cleared in lactic acid. Photomicroscope images were taken with a Canon EOS 750D zoom digital camera (24.2 megapixels) mounted on the Olympus BX51. Raw photos were first stacked with Helicon Focus v. 8.2.0 to obtain the composite images, which were then processed in Adobe Photoshop CC 2020. Scanning electron microscope images (SEM) were taken under high vacuum with a Hitachi S-4800 after critical-point drying and gold-palladium coating. The distribution map was generated with ArcGIS v. 10.2 (ESRI Inc.). All measurements were taken using the Olympus BX51 and are in millimetres. Taxonomic descriptions follow Tong et al. (2020). Type material is deposited in the Shenyang Normal University (SYNU) in Shenyang, Liaoning Province, China (curator: Yanfeng Tong).

The following abbreviations are used in the text and figures: ALE = anterior lateral eyes; ap = apodeme; as = anterior sclerite; bll = blade-like lobe; blp = basal leaf-shaped projection; glo = globular structure; lb = lateral branch; mb = medial branch; nlb = needle-like branch; PME = posterior median eyes; psp = posterior spiracle; sar = sclerotised, recurved arches; slh = small hole; sri = small ridges; tba = transverse bars; tsc = transverse sclerite.

## Taxon treatments

### Trilacuna jimengi

Dai & Tong sp. nov.

3E4361D1-EEFA-5C63-BD56-D1B7EB5FC836

1D70A61B-7E05-4EE9-BF46-AA955474F651

#### Materials

- **Type status:** Holotype. **Occurrence:** catalogNumber: SYNU-F-4861; recordedBy: Jimeng Ma; individualCount: 1; sex: male; lifeStage: adult; occurrenceID: 9AB5A0E1-2740-5342-96DB-8FAB109DF0BF; **Taxon:** scientificName: *Trilacuna jimengi*; order: Araneae; family: Oonopidae; genus: Trilacuna; **Location:** country: China; stateProvince: Yunnan; county: Lincang City; locality: Cangyuan Wa Autonomus County, 500m Northeast of Erma River; verbatimElevation: 1561.05 m; verbatimCoordinates: 23°12′44″N, 99°11′58″E; **Identification:** identifiedBy: Yanfeng Tong; **Event:** samplingProtocol: sifting leaf litter; eventDate: 30/09/2024
- **Type status:** Paratype. **Occurrence:** catalogNumber: SYNU-F-4862-4863; recordedBy: Jimeng Ma; individualCount: 2; sex: female; lifeStage: adult; occurrenceID: 5DFCD329-533F-5A50-A5BA-09A7C7D79AAB; **Taxon:** scientificName: *Trilacuna jimengi*; order: Araneae; family: Oonopidae; genus: Trilacuna; **Location:** country: China; stateProvince: Yunnan; county: Lincang City; locality: Cangyuan Wa Autonomus County, 500m Northeast of Erma River; verbatimElevation: 1561.05 m; verbatimCoordinates: 23°12′44″N, 99°11′58″E; **Identification:** identifiedBy: Yanfeng Tong; **Event:** samplingProtocol: sifting leaf litter; eventDate: 30/09/2024

#### Description

**Figure 1.**
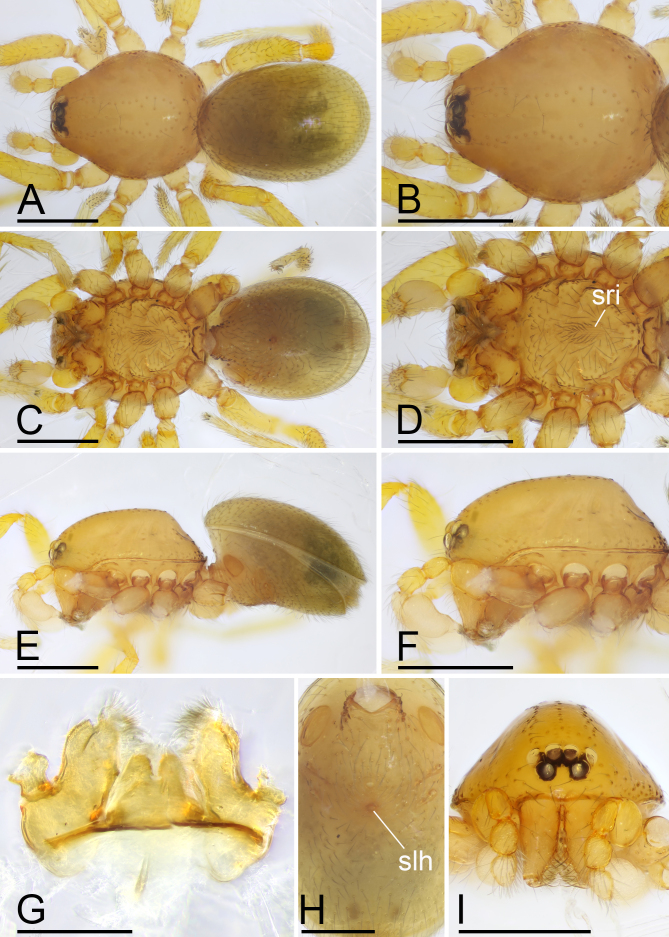
*Trilacuna jimengi* sp. nov., male holotype. **A** habitus, dorsal view; **B** prosoma, dorsal view; **C** habitus, ventral view; **D** prosoma, ventral view; **E** habitus, lateral view; **F** prosoma, lateral view; **G** labium and endites, ventral view; **H** abdomen, ventral view; **I** prosoma, anterior view. Abbreviations: slh = small hole; sri = small ridges. Scale bars: A–F, I = 0.4 mm; G, H = 0.2 mm.

**Figure 2.**
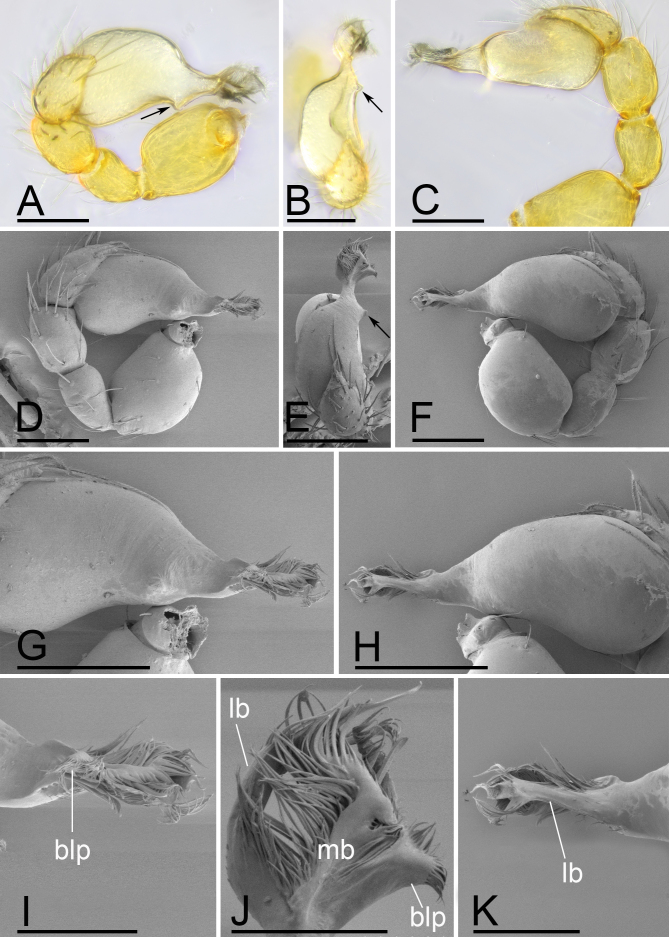
*Trilacuna jimengi* sp. nov., male holotype, light (A-C) and SEM (D-K) images. **A** left palp, prolateral view; **B** left palp, dorsal view, arrows in A, B and E show the small apophysis; **C** left palp, retrolateral view; **D** left palp, prolateral view; **E** left palp, dorsal view; **F** left palp, retrolateral view; **G** bulb, prolateral view; **H** bulb, retrolateral view; **I** distal part of bulb, prolateral view; **J** distal part of bulb, dorsal view; **K** distal part of bulb, retrolateral view. Abbreviations: blp = basal leaf-shaped projection; lb = lateral branch; mb = medial branch. Scale bars: A–H = 0.1 mm; I–K = 0.05 mm.

**Male (Holotype). Body**: habitus as in Fig. 1A, C and E; body length 1.58; yellow. **Carapace**: 0.78 long, 0.64 wide; oval in dorsal view, sides smooth (Fig. 1B and F). **Eyes**: Six eyes, well developed; ALE separated from edge of carapace by 1.1 diameters (Fig. 1I). **Mouthparts**: chelicerae straight; labium rectangular, fused to sternum, anterior margin deeply incised; endites slender, distally only slightly branched (Fig. 1D and G). **Sternum**: surface smooth, sternum covered with many needle-like setae, with several rows of ridges and short and fine setae in median part (Fig. 1D). **Abdomen**: 0.92 long, 0.61 wide; elongated oval in dorsal view, covered with fine setae; booklung covers ovoid (Fig. 1H); sperm pore situated in front of anterior spiracles; posterior spiracles connected by shallow groove; with small hole situated between anterior and posterior spiracles. **Palp**: yellow; 0.51 long (0.16, 0.11, 0.10, 0.14); femur swollen (width/length = 0.75); tibia about as long as patella; bulb kidney-shape, tapering apically, with a small apophysis on subapical region; psembolus complex, with basal leaf-shaped projection (blp), broad median branch (mb) and lateral branch (lb), surrounded by numerous fibre structures (Fig. 2A–K).

**Figure 3.**
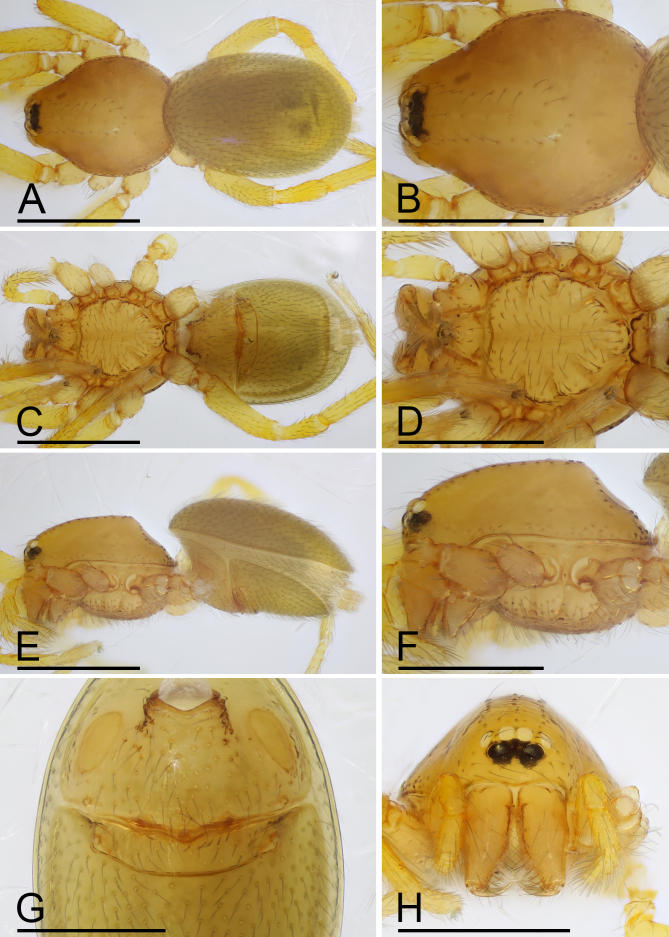
*Trilacuna jimengi* sp. nov., female paratype. **A** habitus, dorsal view; **B** prosoma, dorsal view; **C** habitus, ventral view; **D** prosoma, ventral view; **E** habitus, lateral view; **F** prosoma, lateral view; **G** abdomen, ventral view; **H** prosoma, anterior view. Scale bars: A–F, H = 0.4 mm; G = 0.2 mm.

**Female (paratype, SYNU-F-4862).** Same as male, except as noted. Habitus as in Fig. 3A, C and E. **Body**: 1.55 long. **Carapace**: 0.69 long, 0.58 wide. **Sternum**: without rows of ridges in median part (Fig. 3D). **Abdomen**: 1.87 long, 0.59 wide. **Epigastric area**: middle part of anterior margin of postgastric scutum triangular shape; with recurved, strongly sclerotised arches (sar) anterior to the spiracles (Fig. 3G and Fig. 7a). **Endogyne**: with narrow, transversally elongated sclerite (tsc); with anterior T-shaped sclerite (as) and posterior large ellipsoidal globular structure (glo); transverse bars (tba) with pair of short lateral apodemes (ap) (Fig. 7b).

#### Diagnosis

The new species is similar to *T. wuhe* Tong, Zhang & Li, 2019 in the rows of ridges on central area of sternum, but can be distinguished by the smooth carapace (vs. granulate; cf. Fig. 1B, F, Fig. 3B, F and Tong et al. (2019): figs. 16D, F, 18D and F), the small apophysis on subapical region of bulb (vs. absent;cf. Fig. 2A, E and Tong et al. (2019): figs. 17A and C) and by the large ellipsoidal globular structure of endogyne (vs. small; cf. Fig. 7a, b and Tong et al. (2019): fig. 25B).

#### Etymology

The specific name is named after the collector, Jimeng Ma.

#### Distribution

Known only from the type locality, Yunnan Province, China (Fig. 8).

### Trilacuna nangunhe

Dai & Tong sp. nov.

67575390-6C66-57E4-AEEB-4330EE4BD3AC

74EB42F9-0212-44F3-96EF-460BAD202B6A

#### Materials

- **Type status:** Holotype. **Occurrence:** catalogNumber: SYNU-F-4864; recordedBy: Jimeng Ma; individualCount: 1; sex: male; lifeStage: adult; occurrenceID: 2D62C800-7204-5833-BA28-076DDD92E064; **Taxon:** scientificName: *Trilacuna nangunhe*; order: Araneae; family: Oonopidae; genus: Trilacuna; **Location:** country: China; stateProvince: Yunnan; county: Lincang City; locality: Gengma County, Mengjian Village, Nantianmen; verbatimElevation: 1955.18 m; verbatimCoordinates: 23°38′35.84″N, 99°23′6.96″E; **Identification:** identifiedBy: Yanfeng Tong; **Event:** samplingProtocol: sifting leaf litter; eventDate: 28/11/2024
- **Type status:** Paratype. **Occurrence:** catalogNumber: SYNU-F-4865-4869; recordedBy: Jimeng Ma; individualCount: 5; sex: 2 males, 3 females; lifeStage: adult; occurrenceID: A77A5695-8823-5FB1-81ED-00B694C26C07; **Taxon:** scientificName: *Trilacuna nangunhe*; order: Araneae; family: Oonopidae; genus: Trilacuna; **Location:** country: China; stateProvince: Yunnan; county: Lincang City; locality: Gengma County, Mengjian Village, Nantianmen; verbatimElevation: 1955.18 m; verbatimCoordinates: 23°38′35.84″N, 99°23′6.96″E; **Identification:** identifiedBy: Yanfeng Tong; **Event:** samplingProtocol: sifting leaf litter; eventDate: 28/11/2024
- **Type status:** Paratype. **Occurrence:** catalogNumber: SYNU-F-4870; recordedBy: Jimeng Ma; individualCount: 1; sex: female; lifeStage: adult; occurrenceID: A8C69DA3-FF04-56E8-9976-63DE19C19F14; **Taxon:** scientificName: *Trilacuna nangunhe*; order: Araneae; family: Oonopidae; genus: Trilacuna; **Location:** country: China; stateProvince: Yunnan; county: Lincang City; locality: Gengma County, Banmai Village, Back Mountain of the Management and Protection Station; verbatimElevation: 1538.04 m; verbatimCoordinates: 23°26′24.93″N, 99°16′37.58″E; **Identification:** identifiedBy: Yanfeng Tong; **Event:** samplingProtocol: sifting leaf litter; eventDate: 27/11/2024

#### Description

**Figure 4.**
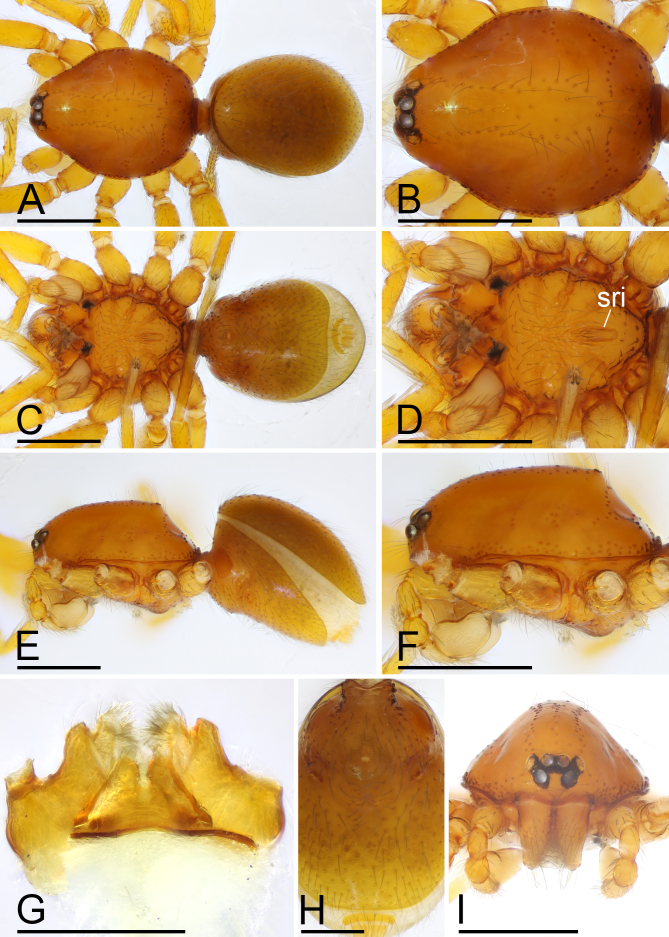
*Trilacuna nangunhe* sp. nov., male holotype. **A** habitus, dorsal view; **B** prosoma, dorsal view; **C** habitus, ventral view; **D** prosoma, ventral view; **E** habitus, lateral view; **F** prosoma, lateral view; **G** labium and endites, ventral view; **H** abdomen, ventral view; **I** prosoma, anterior view. Abbreviation: sri = small ridges. Scale bars: A–F, I = 0.4 mm; G, H = 0.2 mm.

**Figure 5.**
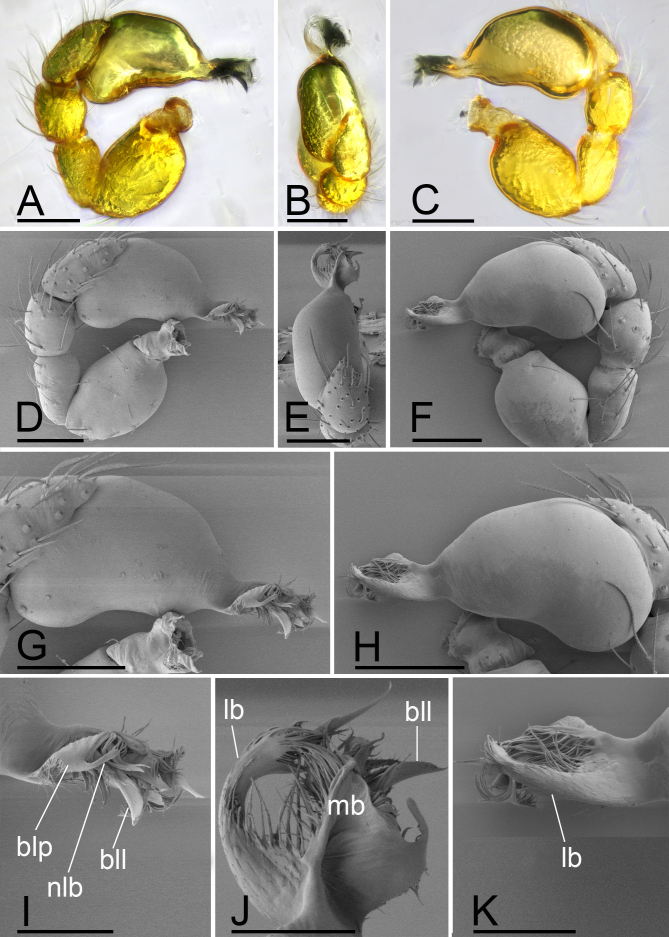
*Trilacuna nangunhe* sp. nov., male holotype, light (A–C) and SEM (D–K) images. **A** left palp, prolateral view; **B** left palp, dorsal view; **C** left palp, retrolateral view; **D** left palp, prolateral view; **E** left palp, dorsal view; **F** left palp, retrolateral view; **G** bulb, prolateral view; **H** bulb, retrolateral view; **I** distal part of bulb, prolateral view; **J** distal part of bulb, dorsal view; **K** distal part of bulb, retrolateral view. Abbreviations: bll = blade-like lobe; blp = basal leaf-shaped projection; lb = lateral branch; mb = medial branch; nlb = needle-like branch. Scale bars: A–H = 0.1 mm; I–K = 0.05 mm.

**Male (Holotype). Body**: habitus as in Fig. 4A, C and E; body length 1.60; yellowish-brown. **Carapace**: 0.8 long, 0.63 wide; oval in dorsal view, sides smooth (Fig. 4B and F). **Eyes**: Six eyes, well developed; ALE largest, PME smallest; ALE separated from edge of carapace by 1.25 diameters (Fig. 4I). **Mouthparts**: chelicerae straight; labium rectangular, fused to sternum, anterior margin deeply incised; endites slender, distally branched (Fig. 4D and G). **Sternum**: surface smooth, covered with many needle-like setae, medial area strongly rugose, posterior area strongly elevated, with several rows of ridges (Fig. 4D and F). **Abdomen**: 0.94 long, 0.6 wide; elongated oval in dorsal view, covered with fine setae; booklung covers ovoid; sperm pore situated at level of anterior spiracles; posterior spiracles not connected by groove (Fig. 4H). **Palp**: yellow; 0.54 long (0.18, 0.11, 0.10, 0.15); femur swollen (width/length = 0.67); tibia about as long as patella; bulb kidney-shape, base strongly swollen ventrally, tapering apically; psembolus complex, with basal leaf-shaped projection (blp), 1 needle-like branch (nlb), 1 blade-like lobe (bll), broad median branch (mb) and lateral branch (lb), surrounded by numerous fiber structures (Fig. 5A–K).

**Figure 6.**
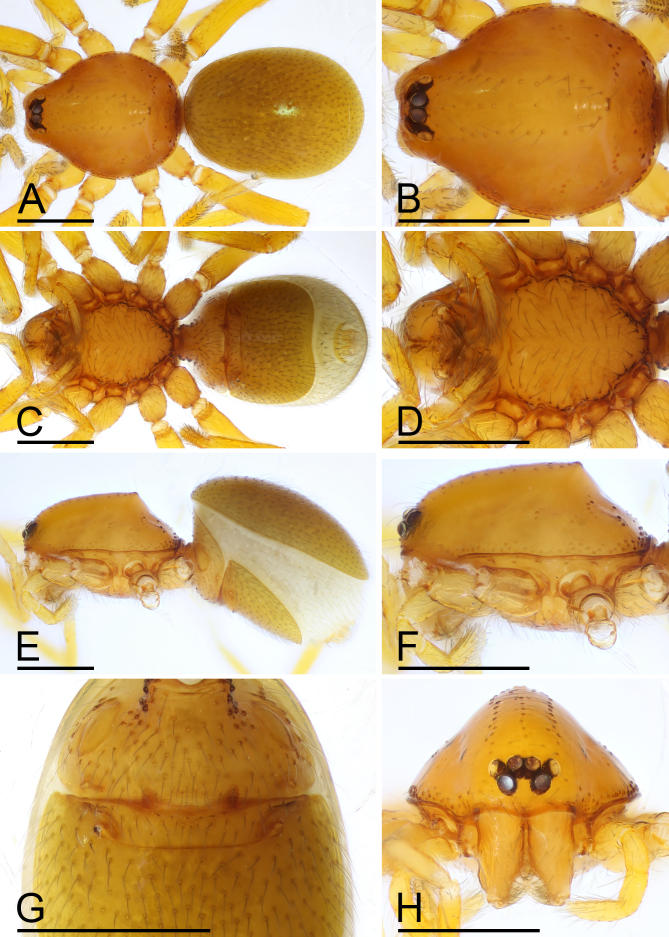
*Trilacuna nangunhe* sp. nov., female paratype. **A** habitus, dorsal view; **B** prosoma, dorsal view; **C** habitus, ventral view; **D** prosoma, ventral view; **E** habitus, lateral view; **F** prosoma, lateral view; **G** abdomen, ventral view; **H** prosoma, anterior view. Scale bar: 0.4 mm.

**Female (paratype, SYNU-F-4865).** Same as male, slightly larger than male, except as noted. Habitus as in Fig. 6A, C and E. **Body**: 1.79 long. **Carapace**: 0.79 long, 0.66 wide. **Sternum**: without rows of ridges in posterior area (Fig. 6D). **Abdomen**: 1.08 long, 0.65 wide. **Epigastric area**: with recurved, strongly sclerotised arches (sar) anterior to the spiracles (Fig. 6G and Fig. 7c). **Endogyne**: with narrow, transversally elongated sclerite (tsc); with anterior T-shaped sclerite (as) and posterior small globular structure (glo); transverse bars (tba) with pair of short lateral apodemes (ap) (Fig. 7d).

#### Diagnosis

The new species is similar to *T. jimengi* sp. nov. in the smooth carapace and the rows of ridges on central area of sternum, but can be distinguished by the male epigastric region lacking a small spot (vs. with a spot; cf. Fig. 4H and Fig. 1H), the psembolus with needle-like branch and blade-like lobe (vs. absent; cf. Fig. 5I and Fig. 1I), the bulb lacking a subapical apophysis (vs. with a small one; cf. Fig. 5D, E and Fig. 1A, E) and the middle part of anterior margin of postgastric scutum being smooth (vs. triangular; cf. Fig. 7c and Fig. 7a).

#### Etymology

The specific name is a noun in apposition taken from the type locality.

#### Distribution

**Figure 8.**
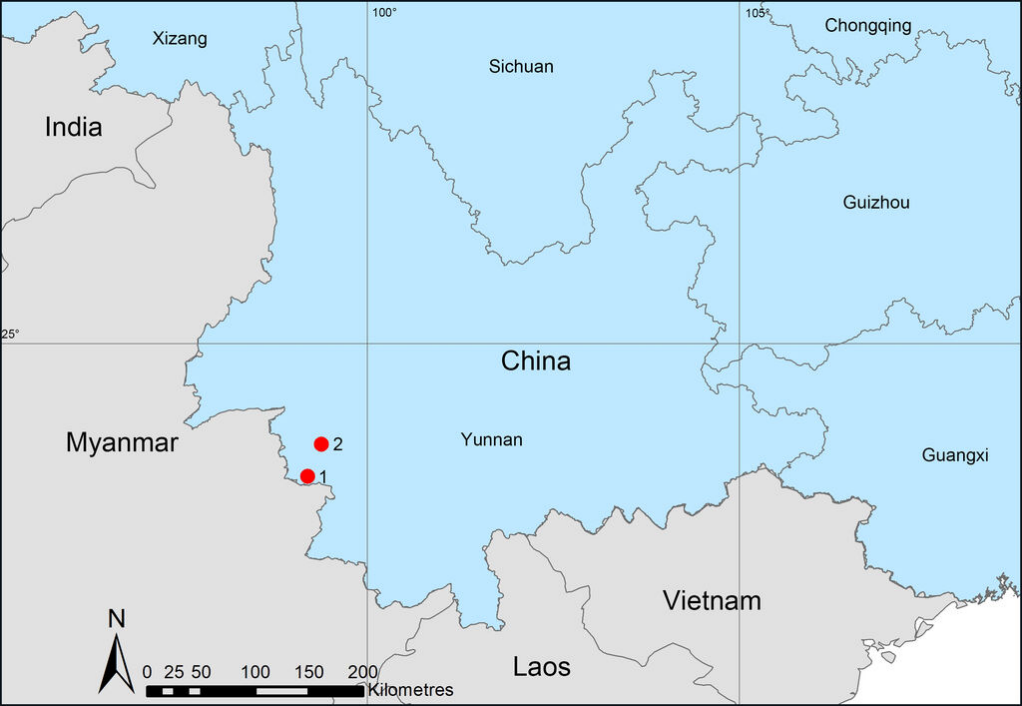
Distribution records of two new species from Nangunhe Nature Reserve, Yunnan, China. **1.** *T. jimengi* sp. nov.; **2.** *T. nangunhe* sp. nov.

Known only from the type locality, Yunnan Province, China (Fig. 8).

## Supplementary Material

XML Treatment for Trilacuna jimengi

XML Treatment for Trilacuna nangunhe

## Figures and Tables

**Figure 7a.**
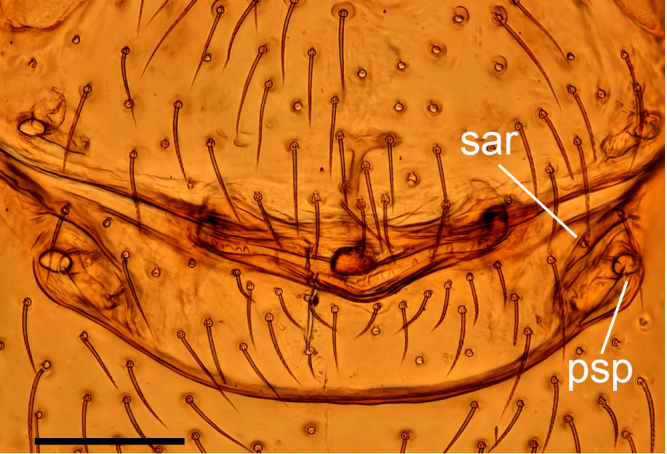
*Trilacuna jimengi* sp. nov., ventral view;

**Figure 7b.**
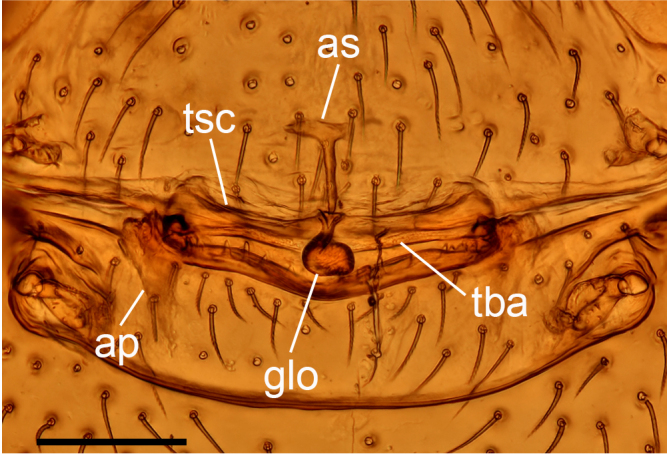
*Trilacuna jimengi* sp. nov., dorsal view;

**Figure 7c.**
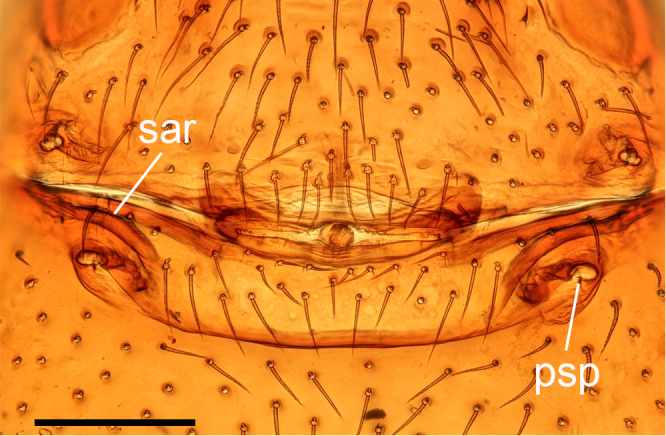
*Trilacuna nangunhe* sp. nov., ventral view;

**Figure 7d.**
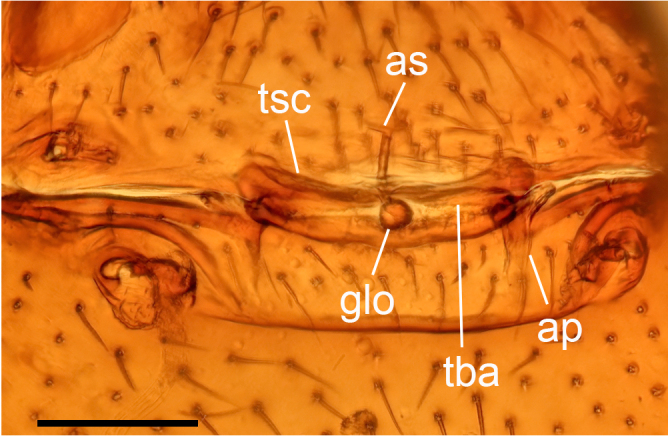
*Trilacuna nangunhe* sp. nov., dorsal view.
